# Rehabilitation of Traumatised Maxillary Anterior Teeth in Children Using Endocrown: A Case Series

**DOI:** 10.7759/cureus.28102

**Published:** 2022-08-17

**Authors:** Pranjali V Deulkar, Sphurti P Bane, Nilesh V Rathi, Nilima R Thosar

**Affiliations:** 1 Pediatric and Preventive Dentistry, Private Practitioner, Nagpur, IND; 2 Pediatric and Preventive Dentistry, Private Practitioner, Mumbai, IND; 3 Pediatric and Preventive Dentistry, Dr. D.Y. Patil Dental College and Hospital, Pune, IND; 4 Pediatric and Preventive Dentistry, Sharad Pawar Dental College and Hospital, Datta Meghe Institute of Medical Sciences, Wardha, IND

**Keywords:** esthetics, pediatric dentistry, endocrown, anterior teeth, dental trauma

## Abstract

Trauma to the anterior teeth has a great impact on the societal and psychosomatic well-being of an individual. Restoring such teeth with a minimally invasive approach is of utmost importance. Endocrown is a monolithic ceramic bonded structure that can be made at dental laboratories for the maintenance of tooth structures. This alternative for endodontically treated teeth turns out to be a viable option in restoring traumatic tooth structures. The steps in its preparation are easy and conservative when compared to conventional crowns with post and core. This article emphasizes two different cases of traumatic anterior teeth in children, successfully dealt with by means of endocrowns fabricated with composite and lithium disilicate prostheses with a 10-month follow-up period.

## Introduction

Trauma to the anterior teeth has a great impact on societal and psychosomatic well-being [1]. When left untreated, a traumatic tooth may show discolouration, ankylosis, and resorption leading to periapical cyst formation. Also, physiological alterations in its composition predispose the tooth to numerous risk factors, such as compromised substrate adhesion, reduced retention, and increased tooth fragility, eventually leading to failure of the prosthesis [2]. Frequently proposed management for such cases is the placement of post and core and crown prostheses [3]. But their aggressive natures have led to concerns amongst clinicians.

Endocrown-type restoration is a monolithic construction, retaining maximum enamel. This type of conservative technique uses the pulp chamber space for retention, thereby favouring effective reconstruction in terms of biomechanics [4,5]. Thus, endocrown was chosen as the treatment modality for the following cases.

## Case presentation

Case 1

**Figure 1:**
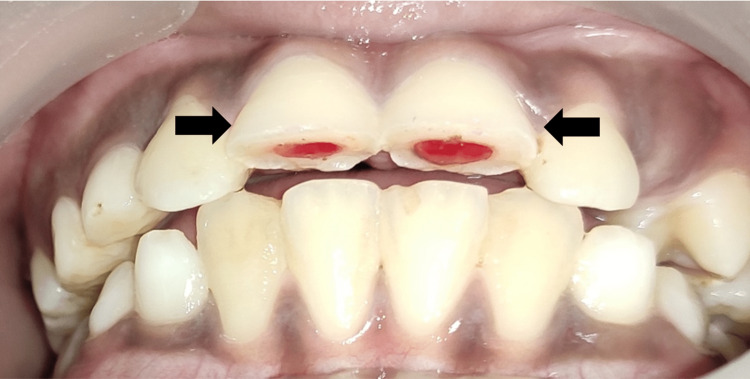
Pre-Operative Image With Fractured 11 and 21 Ellis and Davey's Class III

**Figure 2:**
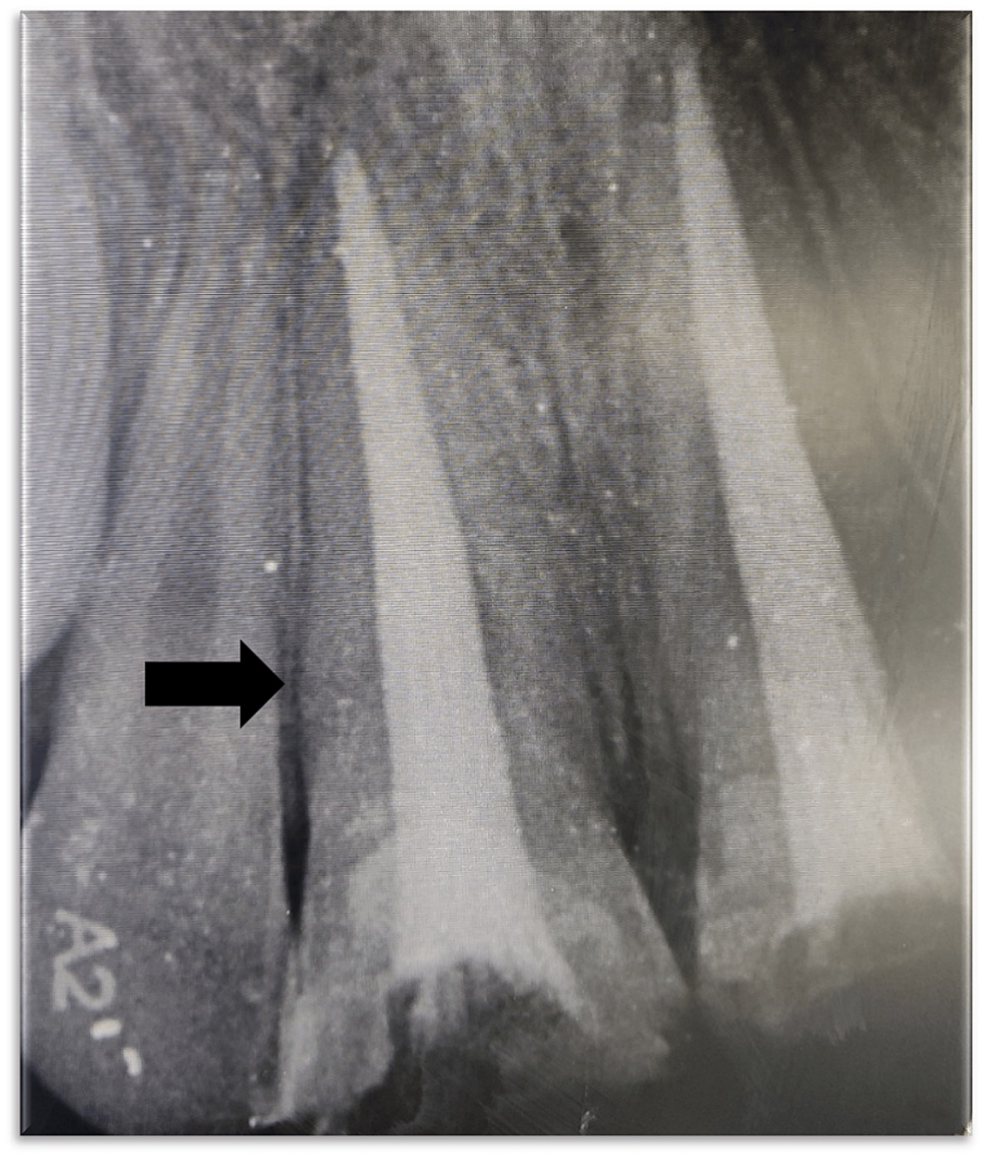
Root Canal Treatment With 11 and 21

**Figure 3:**
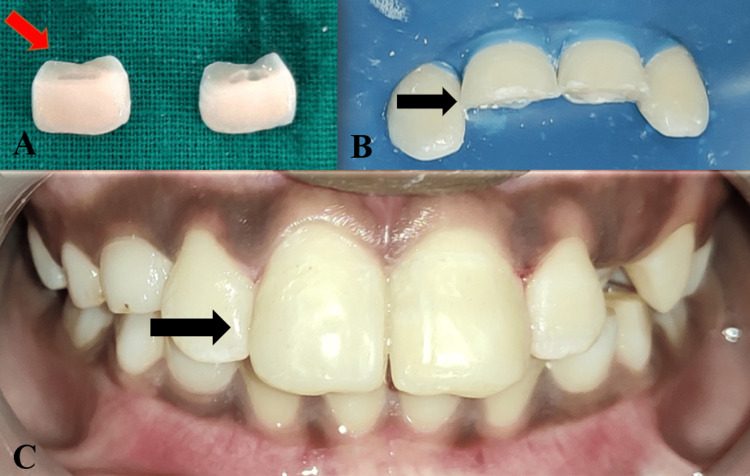
Fragment Re-Attachment With 11 and 21 A: Broken teeth fragments B: Isolation and bevelling of 11 and 21 C: Immediate post-operative image after re-attachment with 11 and 21

A 12-year-old male patient reported to the Department of Pediatric and Preventive Dentistry, complaining of broken teeth in the upper front region of the jaw since a day. The patient experienced sharp, shooting pain. Past medical and dental histories were non-contributory. The separated teeth fragments were carried by the patient's mother in drinking water to the department. Clinical and radiographic examination revealed Ellis and Davey’s Class III fracture with 11 and 21 (Fédération Dentaire Internationale (FDI) notation) (Figure 1). After the formulation of a treatment plan, root canal treatment was performed (Figure 2). This was followed by the reattachment of broken fragments for both teeth (Figure 3).

**Figure 4:**
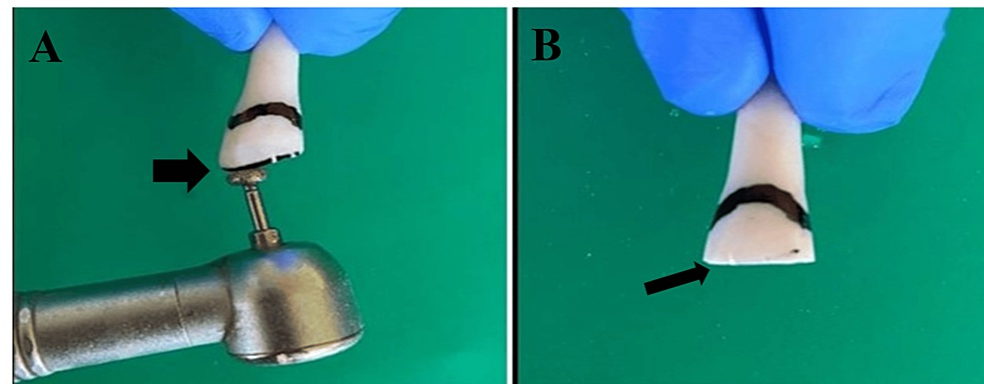
Incisal Levelling With Course Grit Wheel Bur A: Use of course grit wheel bur B: Incisal levelling done

**Figure 5:**
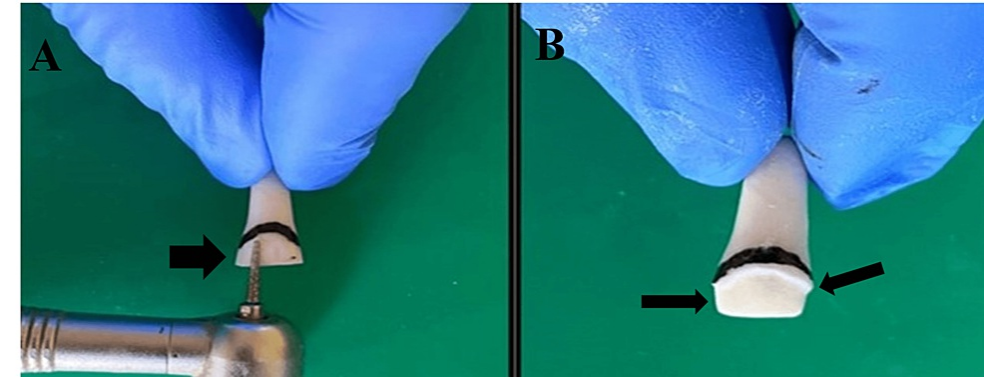
Chamfer Finish Line Preparation With Flat Tapered Fissure Bur A: Use of flat tapered fissure bur for the finish line preparation B: Chamfer finish line preparation can be seen

**Figure 6:**
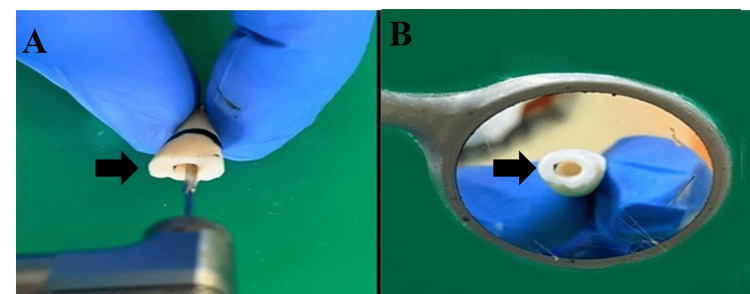
Pulp Chamber Preparation With Peeso Reamer A: Use of the peeso reamer for pulpal space preparation B: Pulp chamber space prepared

A week later the patient revisited the clinic with a broken 11, due to a fall from the bicycle. The patient was evaluated and endocrown preparation with 11 was planned. In this case, Filtek Z350XT (3M-ESPE, Saint Paul, MN) resin composite was selected to fabricate the endocrown at Creative Dental Creations, Nagpur. The preparation has been schematically represented in the following images. First and foremost overall incisal leveling of the fractured segment was carried out using a course grit wheel bur [6] (Figure 4). Then, a labial preparation was done to achieve a chamfer finish line with a flat end tapered bur (Figure 5). This was followed by pulp chamber preparation and removal of the gutta-percha up to 02.00 mm below the level of each orifice with the help of peeso reamers (Figure 6).

**Figure 7:**
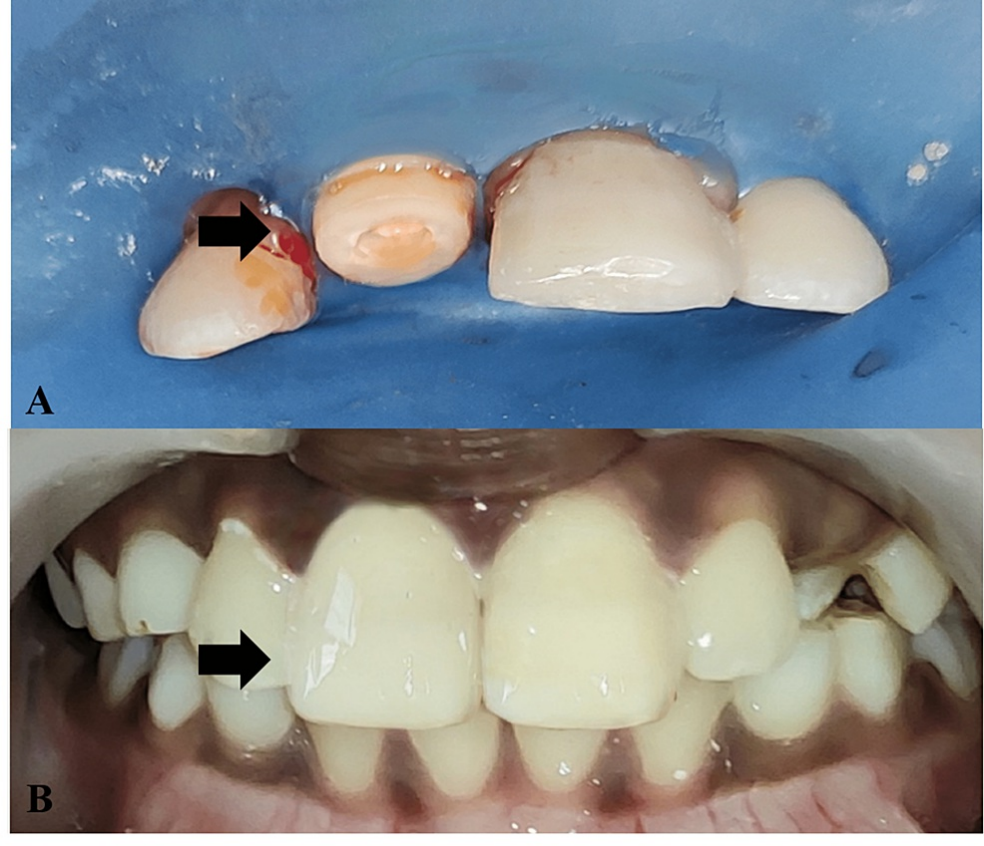
Endocrown Placement With 11 A: Tooth preparation for endocrown with 11 B: Immediate post-operative image after endocrown with 11 placement

Lastly, complete sealing of the coronal orifices and pulp chamber with glass ionomer cement was done [7]. A2 Shade for the prosthesis was then selected. Gingival retraction cord 00 (Ultracord, Dent One Inc, USA) was applied and an impression was made with polyvinyl siloxane material (Aquasil LV, Dentsply DeTrey, Germany) using a putty wash technique and was sent to the laboratory for further processing. Acrylic temporization was done. Filtek Z350XT (3M-ESPE) resin indirect composite endocrown prosthesis was received from the laboratory. In the subsequent appointment, cementation was done using total-etch dual cure resin luting cement (Variolink II, Ivoclar Vivadent) as sufficient enamel was available on the supragingival margins (Figure 7).

**Figure 8:**
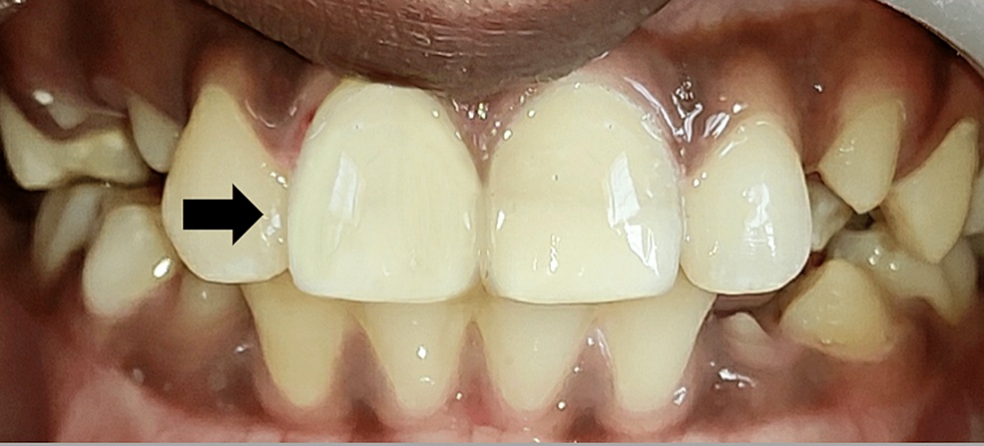
Polishing of the Restoration After 10 Months

After three, six, and the 10-month evaluation, no variations were detected with 11. At the 10-month follow-up, polishing of the restorations was done (Figure 8).

Case 2

**Figure 9:**
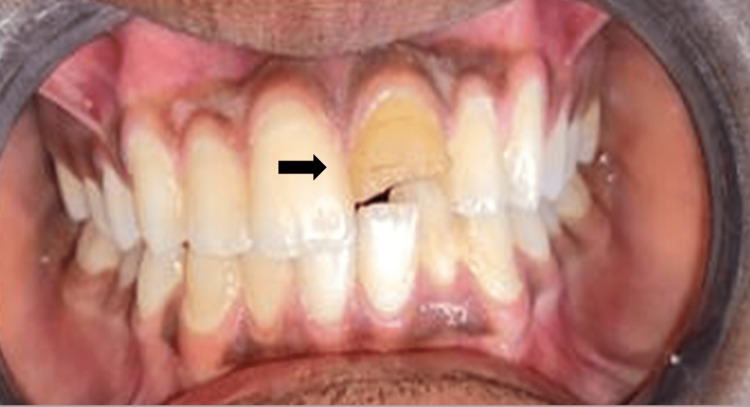
Pre-operative Image With Fractured 21

A 13-year-old male patient visited the Department of Pediatric and Preventive Dentistry, complaining of a broken tooth in the upper front region of the jaw for two months due to a fall while playing. The previous medical history was insignificant but dental history disclosed root canal treatment with 21 followed by direct composite buildup done, six months ago (Figure 9).

**Figure 10:**
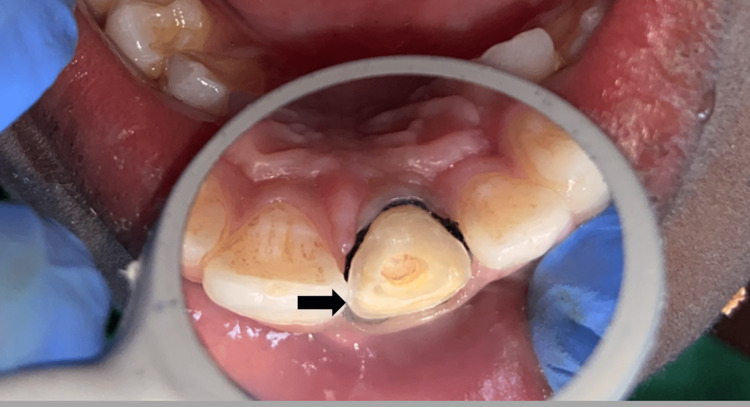
Equigingival Endocrown Tooth Preparation for 21

**Figure 11:**
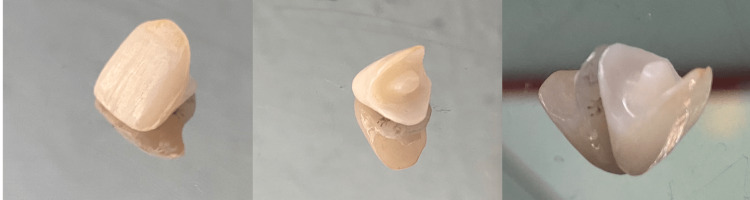
Lithium Disilicate Endocrown With 21

**Figure 12:**
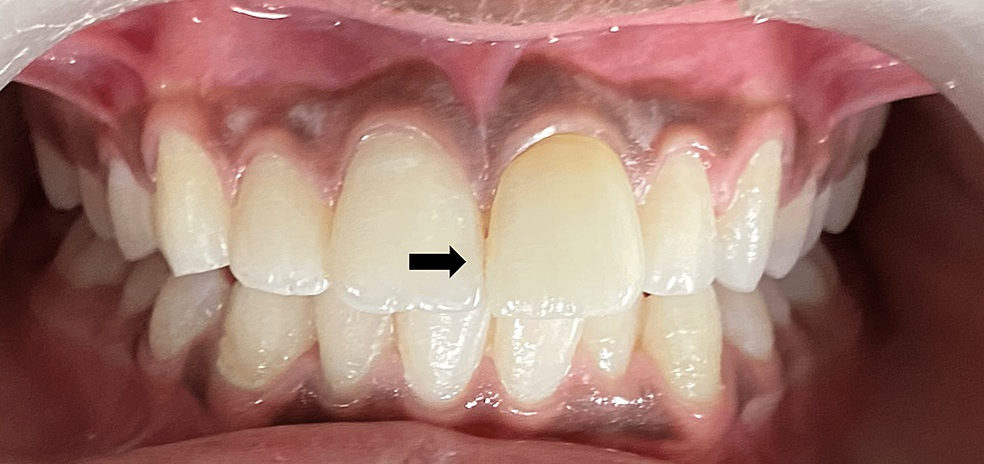
Immediate Post-Operative Image With Endocrown 21

A radiographic investigation was carried out to evaluate the root canal-treated tooth and rule out the presence of any periapical pathologies. After discussing the treatment options, the parents agreed to endocrown restoration made up of lithium disilicate. Steps similar to Case 1 were carried out for the tooth preparation. However, the preparation margin was kept equigingival unlike Case 1, where it was supragingival (as some amount of initial supragingival preparation was done for re-attachment) (Figure 10). Then, the recorded impression was sent to the laboratory for fabrication of a lithium disilicate endocrown (Figure 11). As the margins were equigingival, a less technique-sensitive resin luting cement Rely-X (Unicem, 3M ESPE) was used for cementation on the next visit (Figure 12). Then, the patient was re-evaluated after three months. 

## Discussion

Maxillary central incisors safeguard the posterior teeth during protrusive movements and are engaged in the important task of tearing the food. Thus, proper prosthetic rehabilitation of these teeth if lost is of chief importance. Routinely available treatment options for such teeth are post and core with a crown prosthesis, conventional crown prosthesis, and direct composite build-ups. However, post and core preparation extensively increases the chances of radicular fractures, is more invasive, and has more chances of failure. Also, re-treatment is difficult as retrieval of the post is complicated [8,9]. The conventional crown prosthesis has a greater chance of dislodgement in such cases because of lack of retention, whereas the direct composite build-ups have less strength, and have a tendency to chip off and stain easily [10]. These redundant factors made our search for newer options to restore the teeth. 

Pissis developed a ‘mono-block porcelain technique’ to restore the endodontically treated teeth in a conservative way which was later termed endocrown by Bindl and Mormann [10,11]. Endocrowns maintain the biomechanical integrity of the tooth by sparing the intra-radicular preparation from further compromising the root structure [10]. It preserves as much sound enamel as possible and engages the pulp chamber for macro-mechanical retention. Also, owing to the advent of adhesive dentistry, micro-mechanical retention is achieved by circumferential bonding of tooth structures to the prosthesis [7].

Endocrowns are indicated when there is extreme loss of crown portion, restricted interproximal gap, or in cases short, obliterated, dilacerated, fragile roots. They are contraindicated, where the pulp chamber depth is less than 03.00 mm or cervical margin is less than 02.00 mm wide, and there is the presence of insignificant leftover tooth structure [12].

The decision of choosing the right material for the fabrication of endocrown is of utmost importance. For this, various materials like feldspathic, glass-ceramic, composite resins, CAD/CAM ceramic, and composite resins [13]. The dual shade composite, Filtek Z350XT was selected for endocrown fabrication in the first case, owing to the functional and aesthetic demands. According to research this material demonstrates superior outcomes in terms of compressive, tensile, and flexural strength along with better fracture toughness, wear resistance, low volumetric shrinkage, and good aesthetics [14]. The supremacy of the Z350XT compared to the rest of the composites is easy polishing, good handling properties, and better clinical performance. Gresnigt et al. determined similar fracture strengths between endocrown made of lithium disilicate and indirect composites [9].

CAD/CAM lithium disilicate ceramic was used in the second case as it provides adequate strength and esthetics [15]. Hence, presently it is considered one of the best restorative materials. According to former laboratory trials, ceramic endocrowns showed better fracture strength than composite endocrowns [13,16]. Better bond strength of lithium disilicate to the teeth and lesser bonding interfaces possibly make the endocrowns more resistant when compared to post and core followed by a crown. However, El‑Damanhoury et al. compared the fracture resistance of endocrowns made of feldspathic porcelain, lithium disilicate, and resin composite (Lava Ultimate) and determined that Lava Ultimate composite endocrowns had higher fracture resistance [17]. The difference in these studies may be because of the discrepancy between the structure of the resin composites used as well as the test method and cementation techniques. Endocrown extends a post into the pulp chamber and/or pulp canals as one unit and not a separate post which reduces the number of interfaces [18]. Stresses concentrate in the interface regions. The interfaces of materials have different moduli of elasticity, these regions symbolize the weak points of a restorative system, as this variance influences the stress distribution [19,20].

The reports of these clinical cases designate that the success of endocrown up to a 10-month follow-up time is reasonably good, drawing the conclusion that endocrown restorations are a promising substitute and an added minimally invasive restorative regimen for endodontically treated teeth.

## Conclusions

We experienced two cases of anterior tooth fracture. The management of such fractures was done using endocrown. Endocrown preparation has two main principles, minimal tooth preparation and structure preservation. Endocrown, as a part of minimal intervention dentistry (MID), should be considered as a treatment option to preserve the tooth structure.
